# Studies on Acetone Powder and Purified *Rhus* Laccase Immobilized on Zirconium Chloride for Oxidation of Phenols

**DOI:** 10.1155/2012/375309

**Published:** 2012-03-25

**Authors:** Rong Lu, Tetsuo Miyakoshi

**Affiliations:** Department of Applied Chemistry, School of Science and Technology, Meiji University, 1-1-1 Higashi-mita, Tama-ku, Kawasaki-shi 214-8571, Japan

## Abstract

*Rhus* laccase was isolated and purified from acetone powder obtained from the exudates of Chinese lacquer trees (*Rhus vernicifera*) from the Jianshi region, Hubei province of China. There are two blue bands appearing on CM-sephadex C-50 chromatography column, and each band corresponding to *Rhus* laccase 1 and 2, the former being the major constituent, and each had an average molecular weight of approximately 110 kDa. The purified and crude *Rhus* laccases were immobilized on zirconium chloride in ammonium chloride solution, and the kinetic properties of free and immobilized *Rhus* laccase, such as activity, molecular weight, optimum pH, and thermostability, were examined. In addition, the behaviors on catalytic oxidation of phenols also were conducted.

## 1. Introduction

*Rhus* laccase (EC.1.10.3.2) is a copper-containing glycoprotein occurring in the exudates of lacquer trees. Yoshida [1] first discovered the enzyme in 1883. Since then, many studies of the enzyme have been conducted. However, the results obtained so far in different laboratories frequently show considerable discrepancies. For example, the molecular weight reported varies from 100 to 141 kDa [2–4], and the properties of coppers differ considerably depending on the origin of the laccase preparations [5, 6].

Previously, when the *Rhus* laccase from Japanese lacquer trees was used to oxidize urushiol, the formation of semiquinone radicals, C–C or C–O coupling products, and dibenzofuran compounds were detected [7]. The enzyme laccase, whether obtained from a lacquer tree or fungus, is active in the oxidation of monophenolic compounds such as eugenol and isoeugenol [8]. The laccase-catalyzed oxidation of *O*-phenylenediamine [9], coniferyl alcohol [10], catechol [11], phenylpropanoid [12], and lignocatechol [13] were also demonstrated. Studies of the effects of proteins and polysaccharides in the activities of *Rhus* laccase showed that most proteins and polysaccharides, except laccase proteins, are not only incapable of catalyzing the oxidation of urushiol but can inhibit the activity of laccase to varying extents [14].

Recently, we immobilized *Rhus* laccase from acetone powder obtained from the exudates of lacquer trees grown in the Maoba region, Hubei province of China, on water-soluble chitosan and chitosan microspheres, and their properties were compared with transitional metal (Fe^3+^)-immobilized laccase by chelation [15]. The results showed that, compared with the free *Rhus* laccase, immobilized *Rhus* laccase displayed a lower specific activity but has a similar substrate affinity with improved stability of various parameters, such as temperature, pH, and storage time.

Because lacquer trees are sensitive to the environmental changes of the earth, and the place of the sap production is changed in the composition of the liquid ratio and chemical structure of each component. Thus, in order to investigate the similarities and differences between the famous Chinese Maoba and Jianshi lacquer, in the present paper, we report the isolation and purification of *Rhus* laccases from acetone powder obtained from the exudates of lacquer trees grown in the Jianshi region, Hubei Province of China. In addition, the purified and crude *Rhus* laccases from acetone powder were immobilized on zirconium chloride. After the determination of physical and chemical properties of free and immobilized laccases was carried out, the characteristics of immobilized preparations were then compared using isoeugenol and coniferyl alcohol as substrates to compare their efficiency in catalyzing the oxidation of phenols.

## 2. Materials and Methods

### 2.1. Laccase Assays

#### 2.1.1. Oxygen Consumption in Laccase-Catalyzed Oxidation of Catechol

Laccase was assayed by the oxygen electrode method [16]. The sample chamber (0.6 mL) of the oxygen electrode apparatus was washed several times with deionized water, then three times with 5 mM catechol solution in 0.1 M phosphate buffer solution (pH 7.0: substrate solution), and filled with the substrate solution. When the dioxygen reading was stabilized, the reading scale was adjusted to 100%, then 10 *μ*L *Rhus* laccase solution, 2 mg purified *Rhus* laccase in 10 mL 0.1 M phosphate buffer solution (pH 7.0), was injected into the sample chamber, and the dioxygen consumption rate was recorded. At 30°C, the concentration of dioxygen in buffer is 235 *μ*mol/L due to equilibrium of dioxygen between the air and buffer. Because the volume of sample chamber is 0.6 mL, the water in the sample chamber contains 0.141 *μ*mol of dioxygen. One unit of laccase activity was defined as the amount of laccase required to consume 0.01 *μ*mol of dioxygen min^−1^. After 10 *μ*L of the *Rhus* laccase solution was injected into the system, the consumption of dioxygen min^−1^ was measured as percent concentration (C) of 0.141 *μ*mol dioxygen, and the laccase activity g^−1^ of *Rhus* laccase was calculated according to the following formulation:

(1) $$\begin{array}{cc} & \text{Laccase}\;\text{activity}\;\left(\text{units}\;\min^{-1}{\text{g}}^{-1}\right)\\ & \;\;=\left(\frac{C}{100}\right)\times 0.141\times \left(\frac{1}{0.01}\right)\times 5\times 10^{5}\\ & \;\;=C\times 0.141\times 5\times 10^{5}. \end{array}$$

#### 2.1.2. Laccase-Catalyzed Oxidation of *p*-Phenylenediamine by UV Absorbance

Deionized water was added to a solution of 0.27 g (2.5 mmol)* p*-phenylenediamine in 1 mL 0.2 N HCl until the total volume was 50 mL. Five milliliters of this solution was added to 0.1 M phosphate buffer solution (pH 7.5) at 30°C so that the total volume was 50 mL. The concentration of the resulting solution was then 5 mM. The final solution (3 mL) was placed in a quartz cuvette, and 5 *μ*L of the purified *Rhus* laccase solution (13.14 mg mL^−1^) from Jianshi lacquer sap was added. After stirring with a micro-spatula, the cuvette was placed in a UV spectrophotometer, and the change in absorbance at 336 nm was measured as a function of time [17]. The laccase activity is defined as an increase in the absorbance of a particular absorption band at a particular wavelength per unit time (min) and unit weight of laccase (whether g or mg). If the unit of weight is g, then, it can be expressed as

(2) $$\begin{array}{cc} & \text{Laccase}\;\text{activity}\;\left(\text{units}\;\min^{-1}{\text{g}}^{-1}\right)\\ & \;\;=\frac{\text{Absorbance}\left(\Delta A\right)}{\left[\text{Time}\left(\Delta \min\right)\times \;\text{Amount}\;\text{of}\;\text{laccase}\left(\text{g}\right)\right]}. \end{array}$$

### 2.2. Preparation of Acetone Powder

The exudates (250 g) of a lacquer tree from Jianshi region, Hubei Province of China, were filtered through gauze. Then 1000 mL of acetone was added to the filtrate during mechanical stirring. The insoluble material (acetone powder) was washed with acetone and filtered again. This operation was repeated several times until the filtrate became clear. The resulting acetone powder was then dried at room temperature under vacuum. The yield of acetone powder was 20.6 g. Urushiols were recovered from the combined acetone solutions by removal of the solvent at 40°C under vacuum.

### 2.3. Isolation and Purification of *Rhus* Laccase from Acetone Powder

**Scheme 1:**
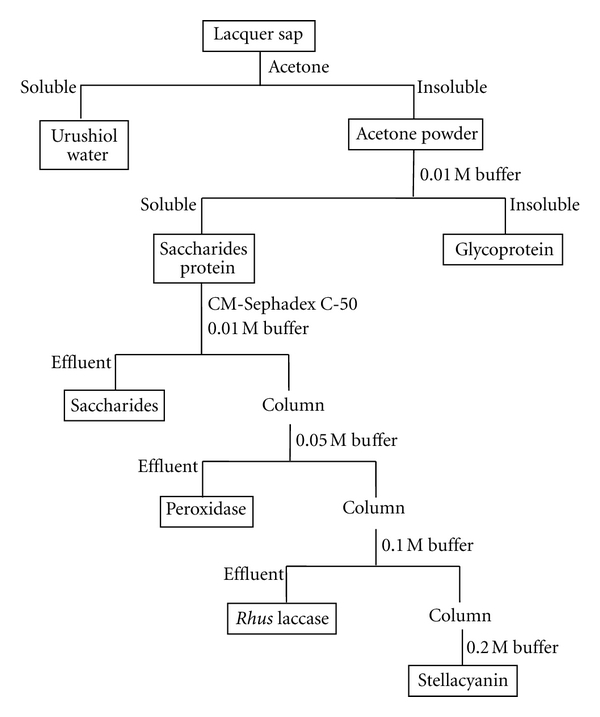
Separation of lacquer sap.

Acetone powder (10 g) was added to 200 mL of 0.01 M potassium phosphate buffer solution (pH 6.0). The resulting mixture was stirred mechanically for 8–12 h in an ice bath. The resulting solution was centrifuged and then filtered to remove any insoluble materials. The filtrate was chromatographed on a CM-Sephadex C-50 column (i.d. 40 mm) prewashed with 0.01 M phosphate buffer solution (pH 6.0) using 0.01 M phosphate buffer solution as the eluent, while being monitored with a UV detector at 280 nm, until no adsorption was observed. The effluent was then transferred to a closed cellulose membrane dialysis tube, which was stirred in 0.01 M phosphate buffer solution in a beaker overnight. The phosphate buffer solution contained mostly polysaccharides. The column was then eluted with 0.05 M phosphate buffer solution (pH 6.0) and monitored with a UV detector at 280 nm until no adsorption was observed. A crude peroxidase solution was obtained by dialysis of the effluent. The column was further eluted with 0.1 M phosphate buffer solution (pH 6.0) and monitored with a UV detector at 280 nm until no adsorption was observed. A crude *Rhus* laccase solution was obtained by dialysis of the effluent. The column was finally eluted with 0.2 M phosphate buffer solution (pH 6.0) and monitored with a UV detector at 280 nm until no adsorption was observed. A crude stellacyanin solution was obtained by dialysis of the effluent. The separation process is shown in Scheme 1.

The crude polysaccharide, peroxidase,* Rhus* laccase, and stellacyanin solutions were separately chromatographed on a DEAE-Sephadex A-50 column using the corresponding buffer solutions to remove yellow components. The resulting effluents containing polysaccharides, peroxidase,* Rhus* laccase, and stellacyanin were then chromatographed individually on a newly prepared CM-Sephadex C-50 column using 0.005 M, 0.025 M, 0.05 M, and 0.1 M phosphate buffer solutions as eluents to obtain crude polysaccharide, peroxidase,* Rhus* laccase, and stellacyanin solutions, respectively [4]. Each effluent was finally desalted and concentrated on a CF25 membrane. The effluents were centrifuged to remove any insoluble materials and then freeze-dried.

### 2.4. Isolation and Purification of *Rhus* Laccases 1 and 2 from the Jianshi Lacquer Exudates

The resulting *Rhus* laccase from the Jianshi lacquer exudates was purified according to the procedure of Reinhammar [4] with a slight modification. The *Rhus* laccase was again chromatographed on a CM-Sephadex C-50 column (i.d. 40 mm). There were two chromatographic bands on the column, a major and a minor band, which were eluted to obtain *Rhus* laccase 1 and *Rhus* laccase 2, respectively.

#### 2.4.1. Determination of Molecular Weights of Purified *Rhus* Laccases 1 and 2 by SDS-PAGE

The molecular weights of *Rhus* laccase 1 and 2 were estimated by SDS-PAGE measurement using myosin (20.5 × 10^4^), *β*-galactosidase (11.6 × 10^4^), bovine serum albumin (8.2 × 10^4^), and ovalbumin (4.7 × 10^4^) as the standard proteins (Prestained SDS-PAGE standards, high range, Bio-Rad's company). Because the concentration of the each standard protein is about 1.25 g/L, the concentrations of *Rhus* laccase 1 and 2 also were about 1.25 g/L.

#### 2.4.2. Optimum pH for Laccase Activity of *Rhus* Laccase 1

Deionized water was added to a solution of 0.27 g (2.5 mmol)* p*-phenylenediamine in 1 mL 0.2 N HCl until the total volume was 50 mL. To 5 mL of the above solution was added 0.1 M Na_2_HPO_4_/KH_2_PO_4_ buffer solution (pH 6.0) at 25°C so that the total volume was 50 mL. The concentration of the resulting solution was 5 mM. The solution (3 mL) was then placed in a quartz cuvette, and 100 *μ*L of the *Rhus* laccase 1 solution (0.1 g mL^−1^) was added. After stirring with a micro-spatula, the cuvette was placed in a UV-spectrophotometer and the increase in the absorbance at 336 nm was measured as a function of time. This experiment was carried out in 0.2 M Na_2_HPO_4_/KH_2_PO_4_ buffer over pH range of 6.5–8.0. In addition, the laccase activity was also assayed in 0.2 M Na_2_HPO_4_/KH_2_PO_4_ buffer solution over a pH range of 7.0–8.5 and Na_2_CO_3_/NaHCO_3_ over a pH range of 8.5-9.5 using 2,6-dimethoxyphenol (DMP) as the substrate.

#### 2.4.3. Thermostability of *Rhus* Laccase 1

The *Rhus* laccase 1 was kept in 0.2 M phosphate buffer solution (pH 6.0) in a temperature range of 40–70°C for 10 min. After rapid cooling, the remaining laccase activity was assayed using *p*-phenylenediamine as substrate as described in Section 2.1.2.

### 2.5. Immobilization of *Rhus* Laccase

#### 2.5.1. Immobilization of Purified *Rhus* Laccase Using Zirconium Chloride as Carrier

To 5 mL of 0.65 M HCl solution was added 0.62 g of ZrCl_4_. The resulting mixture was neutralized with 2 M NH_4_OH solution under a hood and placed in an ice bath. A dropwise solution of 6.9 mg purified *Rhus* laccase in 1.0 mL deionized water was added within 2 h with stirring. The immobilized *Rhus* laccase was filtered and kept in the refrigerator.

#### 2.5.2. Immobilization of Acetone Powder Containing *Rhus* Laccase Using Zirconium Chloride as Carrier

To 5 mL of 0.65 M HCl solution, 0.62 g of ZrCl_4_ was added. The resulting mixture was neutralized with 2 M NH_4_OH solution under a hood and placed in an ice bath. A solution of 1 g acetone powder in 10 mL deionized water was added drop-wise over 2 h with stirring. The immobilized *Rhus* laccase was filtered and kept in the refrigerator.

### 2.6. Kinetics of Immobilized *Rhus* Laccase and Acetone Powder-Catalyzed Oxidation of Phenols

The activity of immobilized laccases was measured spectrophotometrically at 30°C using *p*-phenylenediamine as a substrate; 0.0146 g (0.135 mmol) *p*-phenylenediamine was dissolved in 100 mL 0.02 M pH 6.8 phosphate buffer. The final solution (3 mL) was placed in a quartz cuvette, and the appropriate amount of immobilized laccase was added. After stirring with a microspatula, the cuvette was placed in a UV spectrophotometer and the change in absorbance at 336 nm was measured as a function of time.

### 2.7. Catalysis of Phenols

Isoeugenol (0.5 g) in 10 mL acetone was added to phosphate buffer (0.1 mol/L, pH 7.5, 10 mL). An appropriate amount of each enzyme (see Tables 2 and 3) was added to this substrate solution and was stirred at 30°C for 24 h with aeration. The disappearance of substrate and yields of products was monitored by thin layer chromatography at specific intervals. The solvent of the remaining solution was removed under reduced pressure by evaporation, and the resulting residue was extracted using ethyl acetate, washed with saturated sodium chloride solution, dehydrated and dried over anhydrous sodium sulfate, and then concentrated by evaporation to yield a yellow liquid. This yellow liquid was eluted and purified on silica gel using 3 : 2 (v/v) hexane/ethyl acetate as eluting agent. The products of oxidation were analyzed by gas chromatography (GC) and gas chromatography/mass spectrometry (GC-MS). Oxidation of coniferyl alcohol as catalyzed by the enzymes was similarly performed and purified on silica gel using 1 : 1 (v/v) hexane/ethyl acetate as the eluting agent.

## 3. Results and Discussion

### 3.1. Major Constituents of Jianshi Lacquer Exudates

The exudates of a Chinese lacquer tree (*Rhus vernicifera*) from the Jianshi region, Hubei Province, China, contained about 8.2% of acetone-insoluble components, that is, the acetone powder contained polysaccharides, peroxidase,* Rhus* laccases, and stellacyanin. The remaining acetone-soluble material contained mostly urushiols, although the constituents of the acetone-soluble fraction were not investigated further. The acetone powder was systematically analyzed according to the procedure of Reinhammar [4].

It contained polysaccharides, *Rhus* laccases, peroxidase, and stellacyanin at 25%, 2.1%, 0.13%, and 0.32%, respectively, as summarized in Table 1. The nature of the remaining components is not known.

### 3.2. Purification and Characterization of *Rhus* Laccase 1 and 2

The *Rhus* laccases were purified according to the procedure of Reinhammar [4] with a slight modification. When chromatographed on a CM-Sephadex C-50 column, the *Rhus* laccase was found to contain two chromatographic bands, a major and a minor band, which were denoted laccase 1 and 2, respectively. The purified *Rhus* laccases 1 and 2 were shown to be homogeneous based on polyacrylamide (5%) gel electrophoresis in pH 4.5 buffer solution as previously described [7]. In addition, they each gave only one band on sodium dodecyl sulfate polyacrylamide gel electrophoresis according to the standard method.

**Figure 1:**
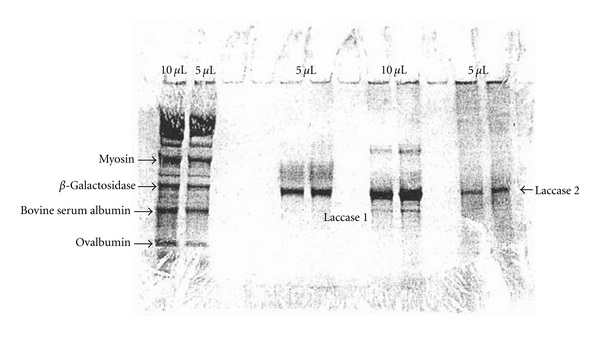
SDS-PAGE results of *Rhus* laccase and standard proteins.

The migrations of *Rhus* laccases 1 and 2 were identical by gel filtration and SDS-PAGE experiments, and the molecular mass of the enzymes was estimated to be 110 kDa (Figure 1). This value is consistent with the reported data for the *Rhus* laccase from Japanese urushi exudates calculated from the copper content. Isoelectric focusing of *Rhus* laccase 1 was conducted on PAGE plates at a pH range of 3–10 using pI markers set (IEF-MIX 3.5–9.3, Sigma Chemical Co.) as the pI indicator. The result showed a major band at pH 8.6 and a very minor band at pH 7.9. A certain asymmetry in the activity curve of *Rhus* laccase was observed in the column isoelectric focusing. Thus, the aforementioned results indicated that *Rhus* laccase 1 had microheterogeneity.

The laccase activity of *Rhus* laccase 1 was determined to be 2.1 × 10^4^ min^−1^ g^−1^ using *p*-phenylenediamine as substrate at pH 7.0. The activity of *Rhus* laccase 2 was determined to be approximately 90% of the *Rhus* laccase 1.

### 3.3. Optimum pH and Thermostability of Crude *Rhus* Laccase in Acetone Powder

**Figure 2:**
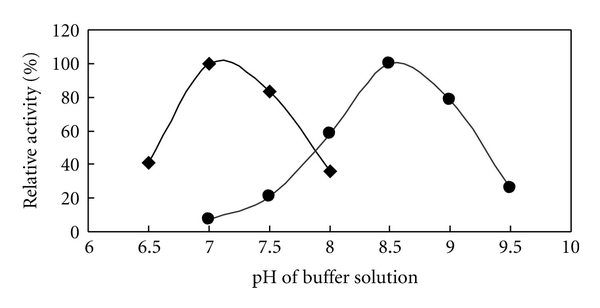
Optimum pH for the crude *Rhus* laccase at 37°C using *p*-phenylenediamine and 2.6-dimethyphenol as substrates: ■: *p*-phenylenediamine; ●: 2.6-dimethyphenol.

 The optimum pH of crude *Rhus* laccase in acetone powder (1 g acetone powder in 20 mL pH 6.86 phosphate buffer) was dependent on the nature of the substrate: pH 7.0 for *p*-phenylenediamine and pH 8.5 for 2, 6-dimethoxyphenol (DMP) at 37°C using 0.2 M Na_2_HPHO_4_/NaH_2_PO_4_ solution over pH range of 6.0–8.0 and Na_2_CO_3_/NaHCO_3_ solution over pH range of 7.0–9.5, as shown in Figure 2. Because of a mixture of *Rhus* laccase 1 and 2, and effect of lacquer polysaccharides or/and other isoenzymes [14], the optimum pH of crude (pH 7.0) and purified (pH 7.5, Figure 4) *Rhus* laccases is slightly difference.

**Figure 3:**
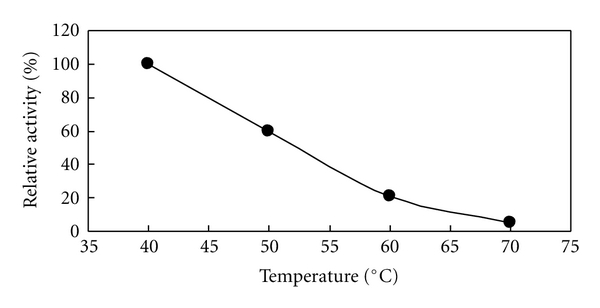
Thermostability of the crude *Rhus* laccase in 0.2 M phosphate buffer at pH 7.0 in the temperature range of 40–70°C.

Because the crude *Rhus* laccase in acetone powder has the highest activity in pH 7.0 at 37°C, the thermostability of them was then examined in 0.2 M Na_2_HPHO_4_/NaH_2_PO_4_ pH 7.0 buffer solution over the temperature range of 40–70°C for 10 minutes, as shown in Figure 3. The crude *Rhus* laccase was heat-resistant vicinity 40°C and almost completely deactivated at 70°C.

### 3.4. Assay of *Rhus* Laccase Activity

The laccase activity of the purified *Rhus* laccase was determined by the oxygen electrode method using catechol, isoeugenol, and other substrates and by UV spectrophotometry with *p*-phenylenediamine as a substrate. The laccase activity of purified *Rhus* laccase 1 was determined to be 3.5 × 10^2^ units min^−1^ g^−1^ using catechol as substrate. Because laccase-catalyzed oxidation of catechol proceeds according to the following reaction equation:


(3)
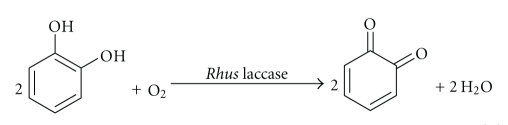



When catechol is used as substrate for assay of the laccase activity, one unit of laccase activity corresponded to the amount of laccase required to reduce 0.01 *μ*mol of dioxygen to 0.02 *μ*mol of water min^−1^. It also corresponds to the amount of laccase required to oxidize 0.02 *μ*mol catechol to 0.02 *μ*mol *o*-quinone min^−1^. When *p*-phenylenediamine was used as the substrate for assay of laccase activity, the change in absorbance at 336 nm was measured as a function of time. The laccase activity is defined as an increase in absorbance of a particular absorption band at particular wavelength with unit time (min) and unit weight of laccase (whether g or mg) [18]. If the unit of weight is the gram, then it can be expressed in units min^−1^ g^−1^, and the laccase activity of purified *Rhus* laccase was determined to be 5.5 × 10^2^ units min^−1^ g^−1^. When isoeugenol was used as the substrate, the laccase activity was determined to be 1.7 × 10^2^ units min^−1^ g^−1^. The difference in activity data may be due to the different water solubilities of substrates and enzyme selectivity.

### 3.5. Immobilization of Purified and Crude *Rhus* Laccase from Acetone Powder

#### 3.5.1. Optimum pH for Immobilized Laccase

**Figure 4:**
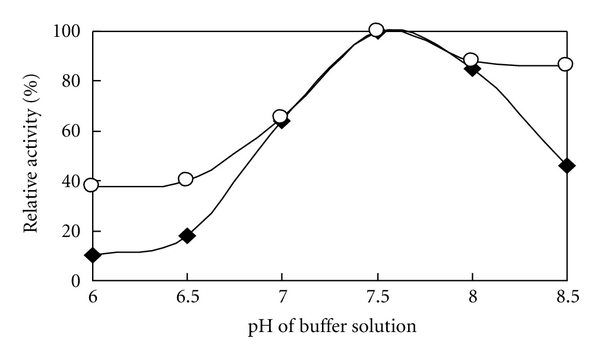
Relationship between pH and activity of *Rhus* laccase, ○: immobilized *Rhus* laccase; ♦: free purified *Rhus* laccase.

The optimum pH for immobilized laccase activity was examined using *p*-phenylenediamine as the substrate at 37°C. The result showed that the optimum pH for immobilized laccase was 7.5. At pH values 6.0, 6.5, and 8.5, the activity of immobilized laccase is more stable and higher than that of the free purified laccase and showed almost the same activity with the free purified laccase at the pH 7.0, 7.5, and 8.0 (Figure 4). It can be considered that because of the interaction between the ZrCl_4_ carrier and laccase, the immobilized laccase formed a stable structure that is less susceptible to the environment, and the activity units calculated with per mg of protein are higher than in free laccase.

#### 3.5.2. Thermostability of Immobilized Laccase

**Figure 5:**
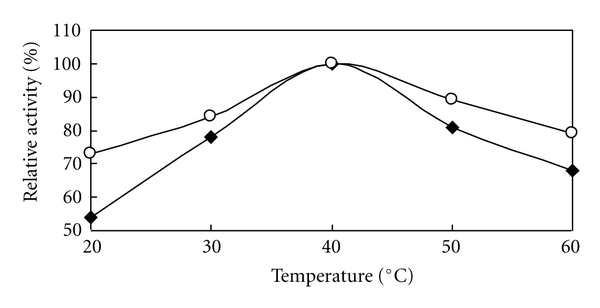
Relationship between temperature and activity of *Rhus* laccase, ○: immobilized *Rhus* laccase; ♦: free purified *Rhus* laccase.

The thermostability of the immobilized laccase was determined using *p*-phenylenediamine as the substrate at pH 7.5. The result showed that the optimum temperature for immobilized laccase is 40°C. In the temperatures at 20, 30 50, and 60°C, the activity of immobilized laccase was more stable and higher than that of the free laccase and showed almost the same activity with the free purified laccase at 40°C (Figure 5). This phenomenon also can be considered due to the stable structure of immobilized laccase.

#### 3.5.3. Effects of Repeated Use of Immobilized Laccase

**Figure 6:**
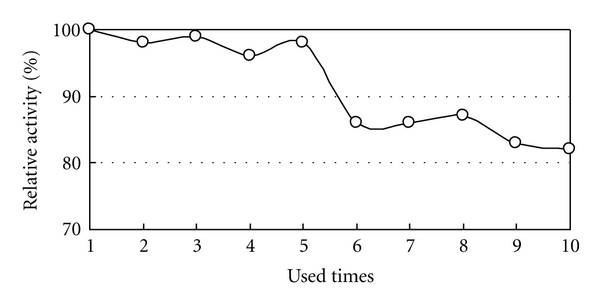
Relationship between repeated used times and activity of *Rhus* laccase.

The effect of repeated use on the immobilized laccase activity was examined using *p*-phenylenediamine as the substrate in phosphate buffer (pH 7.5) at 37°C for 10 minutes and is shown in Figure 6. The relative activity slowly decreased during repeated use, although after 10 uses, it retained over 80% of its initial activity, indicating good potential repeated use efficiency.

### 3.6. Catalytic Oxidation of Isoeugenol

The purified, crude, immobilized purified, and immobilized crude *Rhus *laccases were used to oxidize 0.5 g of each isoeugenol monomers in 30°C. After reacting for 24 h, the solvent was removed by evaporation and extracted with ethyl acetate and washed with saturated NaCl solution. The ethyl acetate extract was dehydrated using anhydrous sodium sulfate. After removing the solvent, a yellow syrupy was obtained. The yellow syrupy product was purified by column chromatography on silica gel (hexane : ethyl acetate = 3 : 2). The overall yields are 0.43, 0.39, 0.49, and 0.36 g respectively, as summarized in Table 2. In Table 2, entries 1, 2, 3, and 4 are the purified, immobilized purified, crude, and immobilized crude *Rhus* laccases, respectively. The reaction solution for purified* Rhus* laccase (entries 1 and 2) was 10 mL 0.1 M phosphate buffer (pH 7.5) mixed with 10 mL acetone and for crude *Rhus* laccase (entries 3 and 4) was 10 mL distilled water mixed with 10 mL acetone.

**Scheme 2:**
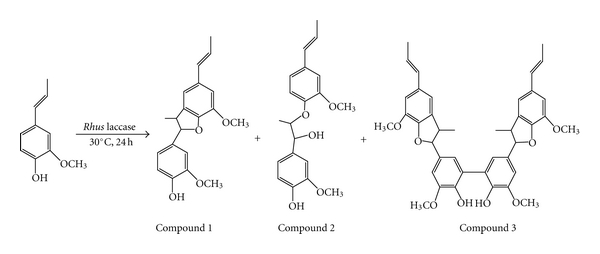
Oxidation of isoeugenol by* Rhus* laccase.

It was found that in the same reaction condition, the ratio of the products is compound 1 > compound 2 > compound 3. The immobilized enzyme (entries 2 and 4) catalyzed more product than the free enzyme (entries 1 and 3), and these results may be due to a stable active site structure of the immobilized enzyme that supports zirconium chloride. In addition, a higher yield was obtained from the crude enzyme catalytic reaction (entries 3 and 4) than the purified enzyme (entries 1 and 2), and this can be considered due to a salt sensitivity of *Rhus* laccase, decreasing the yield of the reaction products in the phosphate buffer. The reaction image is shown in Scheme 2.

### 3.7. Catalytic Oxidation of Coniferyl Alcohol

**Scheme 3:**
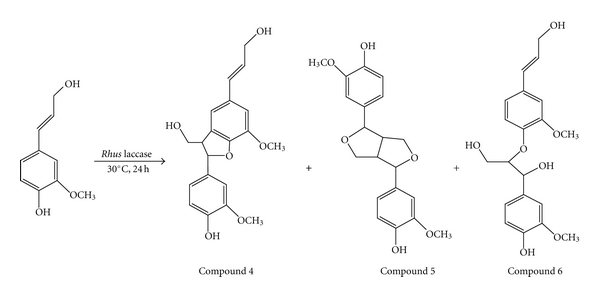
Oxidation of coniferyl alcohol by* Rhus* laccase.

The free crude and immobilized crude *Rhus *laccase from the acetone powder was used to oxidize 0.5 g of each coniferyl alcohol monomer in a mixed solution (10 mL distilled water + 10 mL acetone) at 30°C. After reacting for 24 h, the solvent was removed by evaporation and extracted with ethyl acetate and washed with saturated NaCl solution. The ethyl acetate extract was dehydrated using anhydrous sodium sulfate. After removing the solvent, a light yellow syrupy was obtained. The light yellow syrupy product was purified by column chromatography on silica gel (hexane : ethyl acetate = 1 : 1). The ratio of reaction products is summarized in Table 3, and the reaction image is shown in Scheme 3.

In the same reaction condition, the ratio of the products was compound 4 > compound 5 > compound 6. The yield percentage of each compound catalyzed by the free or immobilized enzyme (entries 1 and 2) was almost the same. Because phosphate buffer was not used in this reaction, no salt sensitivity affected the activity of the free enzyme. In addition, the free enzyme cannot be used again, while the second use of the immobilized enzyme still yielded about 75% coniferyl alcohol dimers (entry 3).

## 4. Conclusion

The *Rhus* laccase in the acetone powder from exudates of Chinese lacquer tree grown in Jainshi, Hubei Province, China, was examined. The crude laccase of the acetone powder was dissolved with phosphate buffer and the laccase purified by a Sephadex column was immobilized with zirconium chloride. The properties of free and immobilized laccase were investigated using *p*-phenylenediamine, isoeugenol, and coniferyl alcohol as substrates. The molecular weight of laccase was estimated to be 110 kDa according to the SDS-PAGE method. The activity of the *Rhus* laccase was determined to be 2.1 × 10^4^ min^−1^ g^−1^ using *p*-phenylenediamine as a substrate at pH 7.0. After immobilization with zirconium chloride by chelation, the immobilized laccase retained over 80% of its initial activity after catalyzing *p*-phenylenediamine 10 times. In the catalytic reaction of isoeugenol to produce isoeugenol dimer, the immobilized enzyme produced more products than the free enzyme, and this may be because of the stable active site structure of the immobilized enzyme that supports zirconium chloride. In the catalytic reaction with coniferyl alcohol to produce dimers, although almost the same yield was observed in the catalyzation by the free or immobilized enzyme, the immobilized enzyme could be used repeatly and about 75% coniferyl alcohol dimer was obtained in the second use as a catalyst.

To summarize, *Rhus* laccase immobilized by chelation using zirconium chloride has many excellent properties. It showed stable activity in organic solvents and water, at various pHs, and reaction temperatures. *Rhus* laccase immobilized with zirconium chloride is an economical enzyme due to its repeated usability and stable activity.

## Figures and Tables

**Table 1:** Yield of the constituents of acetone powder from exudates of Chinese lacquer tree grown in Jain-Shi, Hubei province, China.

Constituents of acetone powder	Yield from 10 g of acetone powder	
	Yield in weight (g)	Yield in percentage(%)
Polysaccharides	2.5	25.0
*Rhus* laccase	0.21	2.1
Peroxidase	0.013	0.13
Stellacyanin	0.032	0.32

**Table 2:** Yield of oxidation of isoeugenol by *Rhus* laccase.

Entry	Yield (g)	Ratio of compounds (%)			
		Isoeugenol	Compound 1	Compound 2	Compound 3
1	0.43	54.2	24.0	18.0	3.7
2	0.39	22.9	47.8	22.3	5.7
3	0.49	7.5	58.2	25.1	9.1
4	0.36	0.6	53.7	30.8	14.8

**Table 3:** Yield of oxidation of coniferyl alcohol by *Rhus* laccase.

Entry	Repeat (times)	Yield (g)	Ratio of compounds (%)			
			Coniferyl alcohol	Compound 4	Compound 5	Compound 6
1	1	0.36	0	60.0	26.1	13.9
2	1	0.31	0	56.3	29.6	14.1
3	2	0.33	24.9	42.8	21.1	11.2
